# Supplementary material for: Modeling the European Neolithic expansion suggests predominant within-group mating and limited cultural transmission
Source: Nat Commun. 2025 Aug 25;16:7905. doi: 10.1038/s41467-025-63172-0 (PMC12379212; doi:10.1038/s41467-025-63172-0)
Supplement: Supplementary file 1 — Supplementary Information [file 41467_2025_63172_MOESM1_ESM.pdf]

Supplementary Information:

Modeling the European Neolithic expansion suggests predominant within-group mating and limited cultural transmission

Troy M. LaPolice[1], Matthew P. Williams[1], Christian D. Huber[1]*

[1]Pennsylvania State University, Department of Biology, University Park, PA 16802, USA

*Correspondence:

Christian D. Huber
Mueller Laboratory, Room 512A
University Park, PA 16802, USA
Email: cdh5313@psu.edu

Table of Contents

Supplementary Notes:

Supplementary Note 1: *Comprehensive description of the symbols and components of the equations in Main Text Fig. 1*

Supplementary Note 2: *Archeological context for outlier individuals with low-levels of EF Ancestry*

Supplementary Note 3: *Statistical Inference of the Cultural Learning Rate*

Supplementary Note 4: *Description of recombination and genome modeling in SLiM*

Supplementary Note 5: *Method for Estimating Age-Specific Mortality Rates and the Equilibrium Fertility Rate*

Supplementary Figures:

Supplementary Figure 1: *Effect of learning rates and within-group mating on front speed*

Supplementary Figure 2: *Effect of within-group mating rates on remaining EF ancestry*

Supplementary Figure 3: *EF ancestry proportion estimations from ancient individuals over distance from farming origin and their geographic region*

Supplementary Figure 4: *Effect of learning rates on remaining EF ancestry*

Supplementary Figure 5: *Quadratic polynomial fit to the log-likelihood values to determine the maximum likelihood estimates*

Supplementary Figure 6: *2D landscape map for complex geography simulations*

Supplementary Figure 7: *Two tested mortality curves*

Supplementary Figure 8: *2D Complex landscape map with topography and Mediterranean dispersal increase*

Supplementary Figure 9: 2D *Complex landscape map with topography, Danube/Rhine dispersal increase and larger Mediterranean dispersal increase*

Supplementary Figure 10: *EF ancestry estimation in ancient individuals and their distance from origin across time periods*

Supplementary Figure 11: *Interaction of learning and within-group mating parameters*

Supplementary Figure 12: *Mean final EF ancestry in the population as a function of the cultural effect given various within-group mating rates*

Supplementary Figure 13: *Interaction of learning and within-group mating parameters without the assumption that children of farmers only become farmers*

Supplementary Figure 14: *Effect of different farmer step sizes on Anatolian farmer ancestry proportion over distance from farming origin*

Supplementary Figure 15: *Ancestry specific marker estimation comparison with qpAdm ancestry estimation*

Supplementary Figure 16: *Geographical and temporal maps of the average qpAdm EF ancestry estimates calculated for each European country for 10 time bins.*

Supplementary Figure 17: *Principal component analysis (PCA) of ancestry in ancient individuals used in fitting.*

Supplementary Figure 18: *Spatial ancestry cline resulting from repeated qpAdm EF ancestry estimation using Anatolian early farmers rather than Balkan early farmers as a proxy for Neolithic EF ancestry.*

References

Supplementary Notes:

Supplementary Note 1: Comprehensive description of the symbols and components of the equations in Main Text Fig. 1

Our reaction-diffusion model describes the spread of farming by tracking three population groups across space: (1) farmers with Early Farming (EF) ancestry (u), (2) farmers with Western Hunter Gatherer (WHG) ancestry (v), and (3) hunter-gatherers with WHG ancestry (w), each measured as population density (individuals per km). The model is defined by a system of partial differential equations, each with three core components: spatial movement (diffusion), population growth, and cultural transmission (learning).

- **Diffusion terms** capture the movement of individuals across space, with group-specific diffusion constants (D_u, D_v, D_w) determining how quickly each population spreads across the landscape.

- **Logistic growth terms** describe how populations locally increase in size over time but are limited by local carrying capacities (K_F for farmers, K_{HG} for hunter-gatherers). Growth slows as population densities approach these environmental limits. The parameters a_u, a_v, and a_w represent the intrinsic growth rates of each population type.

- **Cultural transmission terms** model how hunter-gatherers adopt farming practices from neighboring farmers. The learning rate (f) reflects how frequently this adoption occurs. Adoption also depends on the local density of farmers relative to the total population, meaning transmission is more likely when farmers are common in the area. A preference parameter (γ) controls whether hunter-gatherers are more likely to learn from farmers than from their peers. Specifically, the expression $((u+v)w)/(u+v+\gamma w)$ models the likelihood of hunter-gatherers with WHG ancestry (w) adopting farming practices, depending on the density of farmers ($u+v$) and the preference factor γ.

Together, these three components allow the model to predict how the farming lifestyle—and the associated genetic ancestry—spreads across Europe through a mixture of migration, growth, and learning.

Justification of Logistic Terms

In our model, the logistic terms $1 - (u + v + w) / K_F$ (for farmers) and $1 - (u + v + w) / K_{HG}$ (for hunter-gatherers) assume that farmers and hunter-gatherers compete for shared ecological resources that determine the local carrying capacity. This formulation reflects symmetric density dependence, where all individuals contribute equally to local crowding, resulting in reduced reproduction or increased mortality as the total population density approaches the carrying capacity, K_F or K_{HG}.

This approach contrasts with alternative formulations in the literature where partial niche overlap is modeled using differential weighting of group contributions, such as:

- $\mathbf{1 - (u + v) / K_F, 1 - w / K_{HG}}$ (Aoki et al., 1996; Aoki, 2024)[1,2]
- $\mathbf{1 - (u + v) / K_F - w / K_{HG}}$ (Isern & Fort, 2010)[3]
- $\mathbf{1 - (u + v + c_1 * w) / K_F, 1 - (c_2 * (u + v) + w) / K_{HG}}$ (Cortell-Nicolau et al., 2025)[4]

To evaluate the sensitivity of our results to this assumption, we tested a generalized form with a competition coefficient c (between 0 and 1) controlling the strength of inter-group competition: $1 - (c * w + u + v) / K_F$ for farmers, and $1 - (w + c * (u + v)) / K_{HG}$ for hunter-gatherers. We found that only when c is very small (i.e., when competition or niche overlap is nearly absent) do the ancestry clines differ meaningfully from the baseline model. Accordingly, and for simplicity, we set $c = 1$ in our default formulation (i.e., full competition), consistent with the parameterization used in our Mathematica code.

Supplementary Note 2: Archeological context for outlier individuals with low-levels of EF Ancestry

In our qpAdm[5] analysis we note four individuals—one individual from Spain and three from Germany—passed our filtering threshold but still retained low-levels of EF ancestry (outlier points on Fig. 5b and Supplementary Fig. 4). In Supplementary Fig. 4, points are colored by geographic region. The outlier sample from Spain (NEO646.SG_8413_BP_Spain) is from the site of El Mazo and is one of the earliest instances of Balkan HG ancestry arriving in Iberia[6,7]. The three German individuals—two from Ostorf-Tannenwerder (OST003 and OST002), and one from Blätterhöhle (I1565)—represent crucial evidence of late hunter-gatherer genetic signatures and lifestyles persisting within Neolithic cultural contexts. Blätterhöhle has been shown to have featured a combination of farmer and hunter-gatherer occupation to a relatively late date with individual I1565 (Bla8) previously identified from stable isotopes to be associated with a hunter-gatherer–fisher lifestyle[8]. Inhabitants of Ostorf-Tannenwerder have been previously identified to be one of the last occurrences of high levels of hunter-gatherer-related ancestries prior to the European Bronze Age[9]. Whilst they are in a Funnel Beaker context and adopted some Neolithic cultural elements, their subsistence strategy was consistent with a hunter-gatherer diet[9,10].

Supplementary Note 3: Statistical Inference of the Cultural Learning Rate

This note provides a detailed explanation of the statistical methodology used to infer the cultural learning rate (f) from ancient DNA data. The same approach was used to infer the within-group mating probability parameter.

Supplementary Note 3, Table 1: Notation Used in Statistical Inference.

**Symbol**	**Definition**
f	Cultural learning rate parameter.
i	Index for an individual ancient DNA sample.
N	Total number of ancient DNA samples.
y_i	Observed Early Farmer (EF) ancestry proportion for sample i (from qpAdm[5]).
σ_i	Standard error of the EF ancestry proportion for sample i (from qpAdm[5]).
$\mu_i(f)$	Expected EF ancestry proportion for sample i, predicted by the simulation model given learning rate f.
$L_i(f)$	Likelihood for sample i, given learning rate f.
$LL(f)$	Total log-likelihood of learning rate f across all N samples.
f_k	One of the k discrete learning rates tested in the simulation.
$LL(f_k)$	Total log-likelihood for a specific tested learning rate f_k.

$\hat{f}_{MLE}$	Maximum Likelihood Estimate of the learning rate.
$SE[\hat{f}_{MLE}]$	Standard Error of the Maximum Likelihood Estimate of the learning rate.
a, b, c	Coefficients of the quadratic polynomial $(af^2 + bf + c)$ fit to the log-likelihood values.

A clear definition for every symbol is provided above to act as a legend, facilitating easier comprehension of the subsequent derivations.

I. Overview of the Maximum Likelihood Estimation (MLE) Framework

The primary objective of this statistical analysis is to estimate the cultural learning rate parameter, denoted by f, that best explains the empirically observed spatial pattern of Early Farmer (EF) ancestry, y_i, across N ancient DNA samples (representing different archaeological samples). This estimation is performed within the context of our simulation model of the Neolithic expansion.

Maximum Likelihood Estimation (MLE) is a widely adopted statistical method for estimating the parameters of an assumed probability distribution or a model that generates probabilities[11]. The core principle of MLE is to identify the parameter values that maximize a "likelihood function." The likelihood function, $L(\theta|data)$, quantifies the probability (or probability density for continuous data) of observing the actual empirical data, given a specific value of the parameter θ. The parameter value that maximizes this function is termed the Maximum Likelihood Estimate (MLE), denoted as $\hat{\theta}_{MLE}$. This method is favored due to its desirable asymptotic properties, including consistency (the estimate converges to the true parameter value as sample size increases) and efficiency (it achieves the lowest possible variance among consistent estimators), provided certain regularity conditions are met[11].

In our specific application, we evaluate a predefined set of discrete learning rates, f_k. For each f_k, our simulation model generates a prediction of the expected EF ancestry proportion, $\mu_i(f)$, at each archaeological site i. We then compute the likelihood of having observed the empirical EF ancestry proportions, y_i (each with an associated standard error σ_i), given these model predictions. The learning rate f_k that yields the highest overall likelihood—or an interpolated value derived from a curve fitted to the likelihoods—is considered our $\widehat{f}_{MLE}$.

II. Likelihood of the Learning Rate for an Individual Ancient Sample i

For each individual ancient sample i, the empirical data consist of:

- y_i: The point estimate of the EF ancestry proportion. This value is obtained from qpAdm[5] analysis, as described in the main manuscript.
- σ_i: The standard error associated with the estimate y_i. This, too, is an output of the qpAdm[5] analysis. The qpAdm[5] software provides such estimates and their standard errors, which are crucial for assessing the reliability of the ancestry proportions.

The simulation model, when run with a specific learning rate f, provides:

- $\mu_i(f)$: The expected EF ancestry proportion at the geographic location corresponding to sample i, based on the distance to the farming origin. This prediction arises from the "simple" square landscape simulation model or from more complex landscape models.

To link the observed data with the model predictions, we employ a probabilistic model. We assume that the observed EF ancestry proportion y_i for sample i can be treated as a realization from a normal distribution. The mean of this distribution is considered to be the true EF ancestry at that site, and its standard deviation reflects the uncertainty

inherent in the qpAdm[5] estimation process. We further model the true EF ancestry at site i as being equivalent to our simulation's prediction $\mu_i(f)$, potentially with some deviation.

Combining these considerations, we formally assume that y_i is drawn from a normal distribution with mean $\mu_i(f)$ and standard deviation σ_i (where σ_i is the standard error from qpAdm[5]). This explicitly incorporates the uncertainty of the qpAdm[5] estimate into our likelihood calculation. Mathematically, this assumption is expressed as:

$$y_i \sim N(\mu_i(f), \sigma_i^2)$$

The likelihood of a specific learning rate f, given the data for sample i (i.e., y_i and its uncertainty σ_i), is then given by the probability density function (PDF) of this normal distribution, evaluated at the observed value y_i. The formula for the PDF of a normal distribution $N(\mu, \sigma^2)$ is $PDF(x; \mu, \sigma^2) = \frac{1}{\sigma^2\sqrt{2\pi}} exp(-\frac{(x-\mu)^2}{2\sigma^2})$.

Thus, the likelihood for sample i is:

$$L_i(f|y_i, \sigma_i) = PDF(y_i; \mu_i(f), \sigma_i^2)$$

$$L_i(f|y_i, \sigma_i) = \frac{1}{\sigma_i^2\sqrt{2\pi}} exp(-\frac{(y_i-\mu_i(f))^2}{2\sigma_i^2})$$

This likelihood value, $L_i(f|y_i, \sigma_i)$, quantifies how probable it is to observe the empirical ancestry y_i (with its associated uncertainty σ_i) if the true learning rate were f (which, in turn, dictates the model's prediction $\mu_i(f)$).

The inclusion of the qpAdm[5] standard error σ_i in this formulation is critical. If σ_i is large (indicating a less precise EF ancestry estimate from qpAdm[5]), the squared difference $(y_i - \mu_i(f))^2$ is divided by a larger number $(2\sigma_i)$. This makes the magnitude of the

negative exponent smaller, meaning the likelihood value is less severely penalized for deviations between the observed y_i and the model-predicted $\mu_i(f)$. Consequently, the likelihood function L_i for such a sample will be wider and flatter across different values of f. This effectively down-weights the influence of less certain data points on the overall likelihood function and thus on the final estimate of f. Samples with smaller σ_i (more precise estimates) will have a sharper likelihood function and exert a stronger influence on the parameter estimation. This ensures that more reliable data points contribute more substantially to the inference.

III. Joint Likelihood Across All Samples

To evaluate a given learning rate f using all available data, we combine the likelihoods from individual samples. This requires an assumption about the relationship between the estimation errors for different samples. We assume that the estimation errors for EF ancestry proportions are independent across the N archaeological samples included in the analysis. If the primary factors contributing to estimation error for one sample are unlikely to be systematically correlated with those for another, independence is a reasonable approximation. If errors are independent, the joint probability of observing the entire dataset $Y = (y_1, y_2, ..., y_N)$, given a learning rate f and the set of standard errors $\Sigma = (\sigma_1, \sigma_2, ..., \sigma_N)$, is the product of the individual likelihoods for each sample:

$$L(f|Y, \Sigma) = \prod_{i=1}^{N} L_i(f|y_i, \sigma_i)$$

For reasons of numerical stability and mathematical convenience, it is standard practice to work with the natural logarithm of the likelihood function, known as the log-likelihood ($LL(f)$). Using the property of logarithms that log(A×B)=log(A)+log(B), the log-likelihood becomes a sum:

$$LL(f|Y, \Sigma) = \sum_{i=1}^{N} log(L_i(f|y_i, \sigma_i))$$

Working with the log-likelihood offers several practical advantages. Firstly, maximizing $LL(f)$ with respect to f is equivalent to maximizing $L(f)$ because the logarithm is a monotonically increasing function. Secondly, in computational contexts, summing log-likelihoods prevents arithmetic underflow. Individual likelihood values L_i can be very small numbers. The product of many such small numbers can become exceedingly small, potentially falling below the representational limits of standard computer floating-point arithmetic.

IV. Maximum Likelihood Estimate (MLE) of the Learning Rate ($\widehat{f}_{MLE}$)

The procedure for obtaining the MLE of the learning rate, $\widehat{f}_{MLE}$, involves several steps:

1. Grid Search: A discrete set of plausible learning rates, denoted f_k, is defined. In our study we specified 16 learning rates, ranging from 0 to 0.005 in increments of 0.0003. This range and increment define the grid over which the likelihood function is explored.
2. Simulation and Likelihood Calculation: For each learning rate f_k in this predefined set:
   - The "simple" square (or a complex) landscape simulation model is executed with $f=f_k$. This yields the expected EF ancestry proportions, $\mu_i(f)$, for all N ancient samples (e.g., the blue line in Fig. 5B).
   - The total log-likelihood, $LL(f_k)$, is then calculated using the formula derived in Section III. This process results in a set of 16 pairs of $(f_k, LL(f_k))$ values.

3. Quadratic Approximation: To determine the $\widehat{f}_{MLE}$, which may lie between the discretely tested f_k values, and to facilitate the estimation of its standard error, a quadratic polynomial is fitted to the calculated $(f_k, LL(f_k))$ points. This approximation takes the form:

$LL(f) \approx af^2 + bf + c$

The coefficients (a, b, c) of this polynomial are estimated using a least-squares fitting procedure applied to the $(f_k, LL(f_k))$ data points. The use of quadratic approximations for log-likelihood functions is a common technique in MLE, often justified by the asymptotic normality of MLEs, where the log-likelihood surface near the maximum resembles a quadratic form[11].

4. Determining $\hat{f}_{MLE}$: The value of f that maximizes this fitted quadratic function is taken as the MLE of the learning rate, $\hat{f}_{MLE}$. The maximum of a quadratic function $af^2 + bf + c$ occurs where its first derivative with respect to f is zero:

   $\frac{dLL(f)}{df} = 2af + b = 0$

   Solving for f yields the MLE:

   $\hat{f}_{MLE} = -\frac{b}{2a}$

The quadratic fit serves two primary purposes. Firstly, it allows for interpolation between the discrete points of the grid search, potentially yielding a more precise estimate of the maximum of the true (but computationally expensive to fully map) log-likelihood function. Secondly, it provides a simple analytical form whose derivatives are easily calculated, which is essential for estimating the standard error of $\hat{f}_{MLE}$, as discussed in the next section. The rationale for a quadratic approximation is rooted in the theoretical behavior of log-likelihood functions, which, under fairly general conditions and with sufficient data, tend to be approximately quadratic in the vicinity of their maximum[11].

V. Standard Error Estimation for the Learning Rate Parameter ($\hat{f}_{MLE}$)

The standard error of the MLE, $SE[\hat{f}_{MLE}]$, quantifies the precision of the estimate. Its calculation is based on the asymptotic properties of MLEs and the concept of Fisher Information. A fundamental result in MLE theory states that the variance of an MLE

(e.g., $Var[\hat{f}_{MLE}]$) can be approximated by the inverse of the Fisher Information, $I(f)$, evaluated at the estimate ($\hat{f}_{MLE}$)[11]:

$$Var[\hat{f}_{MLE}] = 1/I(\hat{f}_{MLE})$$

The Fisher Information $I(f)$ can be defined as the negative expectation of the second derivative of the log-likelihood function: $I(f) =- E[\frac{d^2LL(f)}{df^2}]$. In practice, particularly when an analytical expectation is difficult or when dealing with a specific dataset, the observed Fisher Information is often used. This is defined as the negative of the second derivative of the log-likelihood function, evaluated at the MLE:

$$I_{obs}(\hat{f}_{MLE}) =- (\frac{d^2LL(f)}{df^2})|_{f=\hat{f}_{MLE}}$$

Therefore, an approximation for the variance of the MLE is:

$$Var[\hat{f}_{MLE}] \approx \frac{1}{-(\frac{d^2LL(f)}{df^2})|_{f=\hat{f}_{MLE}}} =- ((\frac{d^2LL(f)}{df^2})|_{f=\hat{f}_{MLE}})^{-1}$$

The standard error, $SE[\hat{f}_{MLE}]$, is the square root of this variance:

$$SE[\hat{f}_{MLE}] = \sqrt{- ((\frac{d^2LL(f)}{df^2})|_{f=\hat{f}_{MLE}})^{-1}}$$

This formulation is common in applied MLE[11].

In our specific case, we have approximated the log-likelihood function with the quadratic polynomial $LL(f) \approx af^2 + bf + c$, derived in Section IV.

The first derivative of this approximation is $\frac{dLL(f)}{df} \approx 2af + b$.

The second derivative is constant: $\frac{d^2 LL(f)}{df^2} \approx 2a$.

This value, $2a$, is our estimate of the second derivative of the log-likelihood function at its maximum (since the second derivative of a quadratic is constant, it applies at the maximum as well).

Substituting $2a$ into the formula for the standard error leads to:

$$SE[\hat{f}_{MLE}] = \sqrt{-\frac{1}{2a}}$$

This method directly connects the precision of the estimate to the "curvature" or "sharpness" of the log-likelihood peak. A large negative value of 'a' (i.e., a large magnitude for 'a') signifies a sharply curved peak. This leads to a smaller value for $-\frac{1}{2a}$ and thus a smaller standard error, indicating higher precision in the estimate of f. Conversely, if 'a' is a small negative number (small magnitude), the peak is flatter, resulting in a larger standard error and indicating lower precision. This is intuitively sound: a flat likelihood surface implies that a wider range of parameter values are almost equally plausible given the data, leading to greater uncertainty in the estimate.

Supplementary Note 4: Description of recombination and genome modeling in SLiM

We use the "crossover breakpoints" recombination model in SLiM[12], which is SLiM's standard method for simulating genetic recombination via gametic crossing over[13]. In this model, recombination events are introduced during simulated meiosis by specifying a recombination rate per base pair. We chose a rate of 1 centimorgan per megabase (1 cM/Mb), which reflects the average recombination rate across the human genome[14]. This value corresponds to a 1% chance of a crossover event occurring during meiosis per one million base pairs. Accordingly, this leads to a 1% chance of crossover between our marker mutations that we assume to be one million base pairs apart from each other. As described in the SLiM manual[13], this recombination rate is used when generating parental gametes to randomly place crossover breakpoints along the chromosome. In SLiM[12], each individual carries two homologous chromosomes, and during reproduction, one recombinant chromosome is passed to the offspring, formed by copying segments from the two parental homologs with recombination.

To generate a gamete, SLiM[12] begins copying from one of the two parental homologous chromosomes (i.e., one of the two copies of a chromosome that each individual inherits—one from each parent). Upon reaching a recombination breakpoint, it switches to copying from the other homolog. Thus, these breakpoints simulate crossover events in meiosis, in which genetic material is exchanged between the parental homologs. The result of this process is a recombined haploid gamete, composed of segments inherited from both parental homologs. SLiM[12] then uses these gametes from each parent to assemble the diploid genome of the offspring. SLiM treats this sequence as embedded in a continuous chromosome of defined physical length–in our case, 247 megabases. Therefore, recombination in SLiM[12] results in long contiguous blocks of markers inherited from one or the other parent, as observed empirically in real genomes.

Lastly, of note, for simplicity our model does not assume male or female individuals, thus an individual can, in theory, mate with any other individual with the offspring number being drawn from a Poisson distribution (offspring number ~ Poisson(λ = 0.1)).

Supplementary Note 5: Method for Estimating Age-Specific Mortality Rates and the Equilibrium Fertility Rate

Age-Specific Mortality Rates

We used published age-at-death data derived from Neolithic skeletal remains, binned into discrete yearly age categories (see Supplementary Data File 3; Supplementary Data File 4). These data reflect the relative frequency of individuals who died in each age year and can be interpreted as a stable age-at-death distribution under the assumption of demographic equilibrium. To estimate age-specific mortality rates from this distribution, we followed these steps:

Step 1: Normalize the age-at-death counts to get a probability distribution that sums to 1 across all age bins.

Step 2: Estimate the conditional probability of death in each age bin, given survival to that bin. This is calculated as: $q_x = d_x / (d_x + d_{x+1} + d_{x+2} + ...)$

where:

- q_x is the probability of dying in age bin x, given survival to the start of that bin
- d_x is the number of observed deaths in age bin x
- the denominator is the sum of all deaths in age bin x and older

Step 3: Apply these probabilities as age-specific mortality rates in the agent-based model. These rates are used identically for both farmers and hunter-gatherers.

This approach approximates an "equilibrium" mortality schedule under the assumption of a stable age distribution and no strong age-related preservation bias. While simplified, it provides a plausible mortality pattern consistent with Neolithic skeletal data.

Equilibrium Fertility Rate

To ensure demographic equilibrium in our agent-based model, we derived a fertility rate that balances mortality such that the population remains stable over time (i.e., zero net growth).

Step 1: Using the observed age-at-death distribution from Neolithic skeletal data (see Supplementary Data File 3; Supplementary Data File 4), we estimated the probability of surviving to each age, denoted as s_x. This was calculated as:

s_x = (number of individuals who survived to age x) divided by (total number of individuals)

Here, the number surviving to age x is computed as the sum of all deaths in age x and older. These survival probabilities approximate the age structure of a stable population under the observed mortality pattern.

Step 2: We define the reproductive age range as individuals aged 12 and older, consistent with assumptions in anthropological demography for early farming populations.

Step 3: Under equilibrium conditions, each individual must, on average, produce one surviving offspring to replace themselves. Therefore, the equilibrium fertility rate f_r is calculated as:

f_r = 1 / (sum of s_x for $x \geq 12$)

This gives the per-individual per-year fertility rate required to maintain a stable population size, assuming all mature individuals have equal fertility and mortality remains constant over time. In our case, for the mortality rates in Supplementary Data File 3, the sum of survival probabilities for ages 12 and up is approximately 10, leading to:

$f_r = 1 / 10 = 0.1$

Step 4: This fertility rate is applied in the simulation by drawing the number of offspring from a Poisson distribution with rate (λ) = 0.1. This is done for each individual aged 12 or older who has a suitable mating partner. This implementation ensures that, on average, births and deaths balance out, maintaining demographic equilibrium over time.

Supplementary Figures:

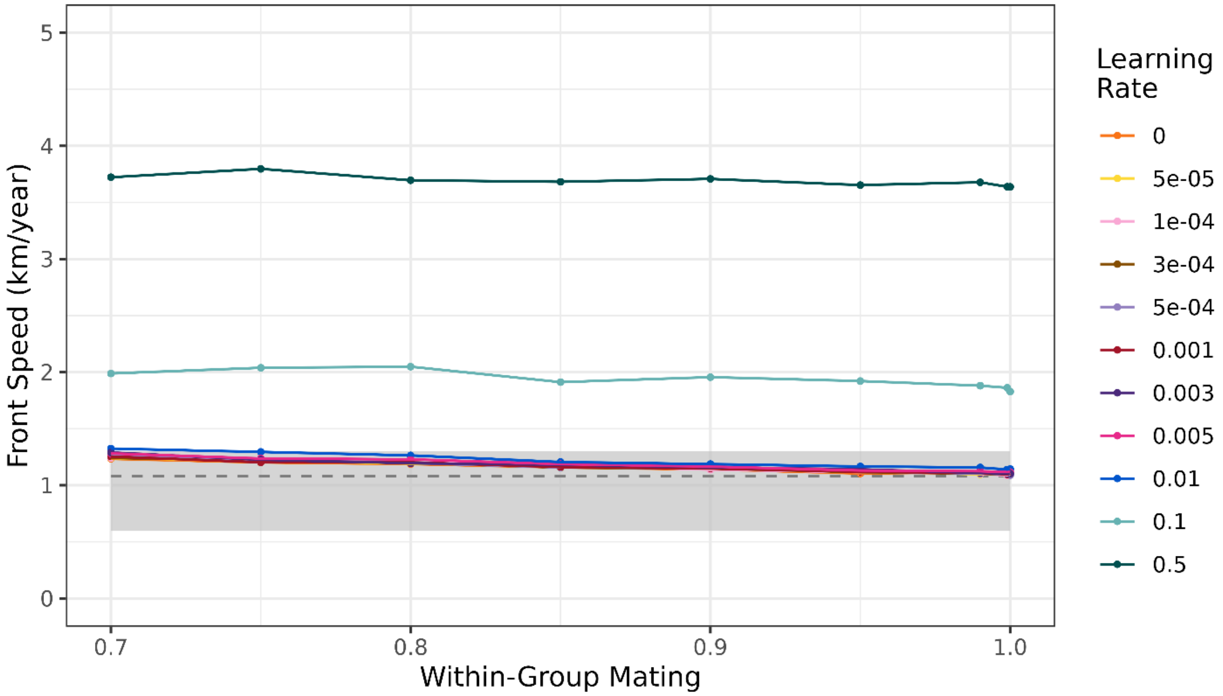

Supplementary Figure 1: Effect of learning rates and within-group mating on front speed

Effect of within-group mating and different learning rates on front speed, given a fixed step size of 5 km. For comparisons, the gray shaded box illustrates the front speed range of 0.6-1.3 km/yr estimated by Pinhasi et al. (2005)[15] and the dashed line represents front speed of 1.08 km/yr predicted by Ammerman and Cavalli-Sforza (1971)[16]. This supplementary plot shows additional very high learning rates not shown in Fig. 3C (0.1, 0.5 per year) that are required to affect front speed.

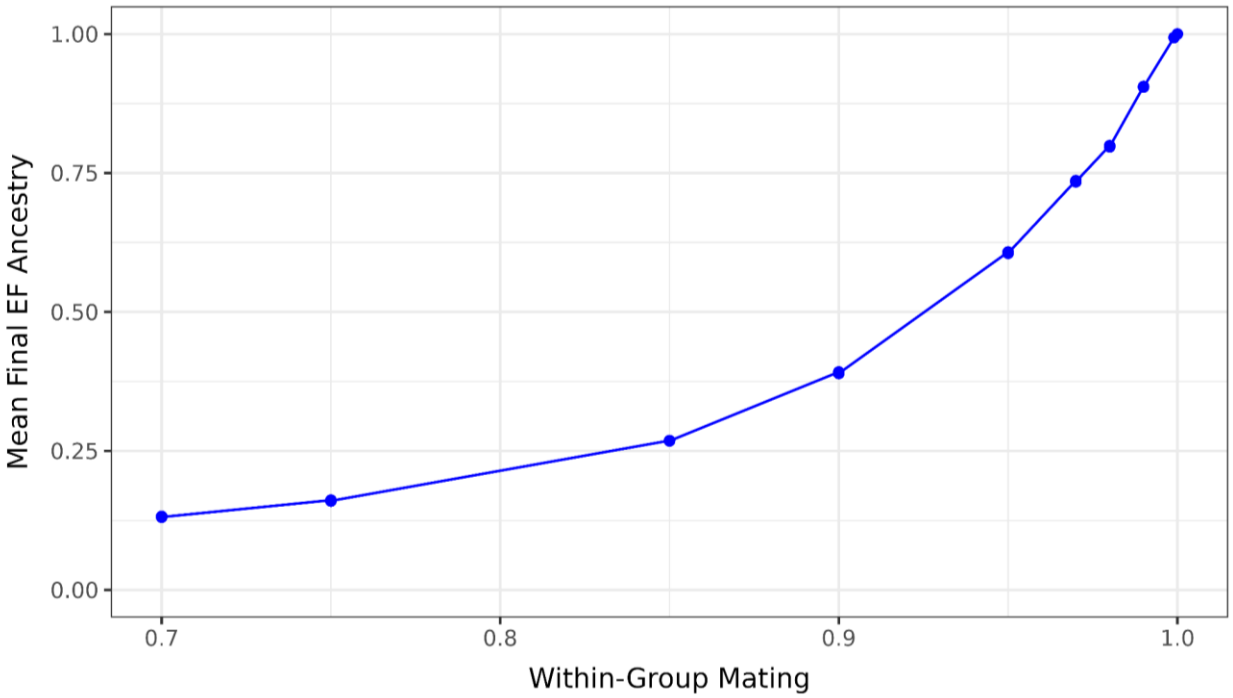

Supplementary Figure 2: Effect of within-group mating rates on remaining EF ancestry

10 simulated within-group mating rates used for fitting empirical data and their effect on the proportion of EF ancestry in the population following the expansion, when farming has become ubiquitous. Figure shows the mean proportion of EF ancestry in the population across the entire landscape. All 10 runs assume zero peer-to-peer learning with only parent-to-child vertical cultural transmission. All runs used the simple square landscape.

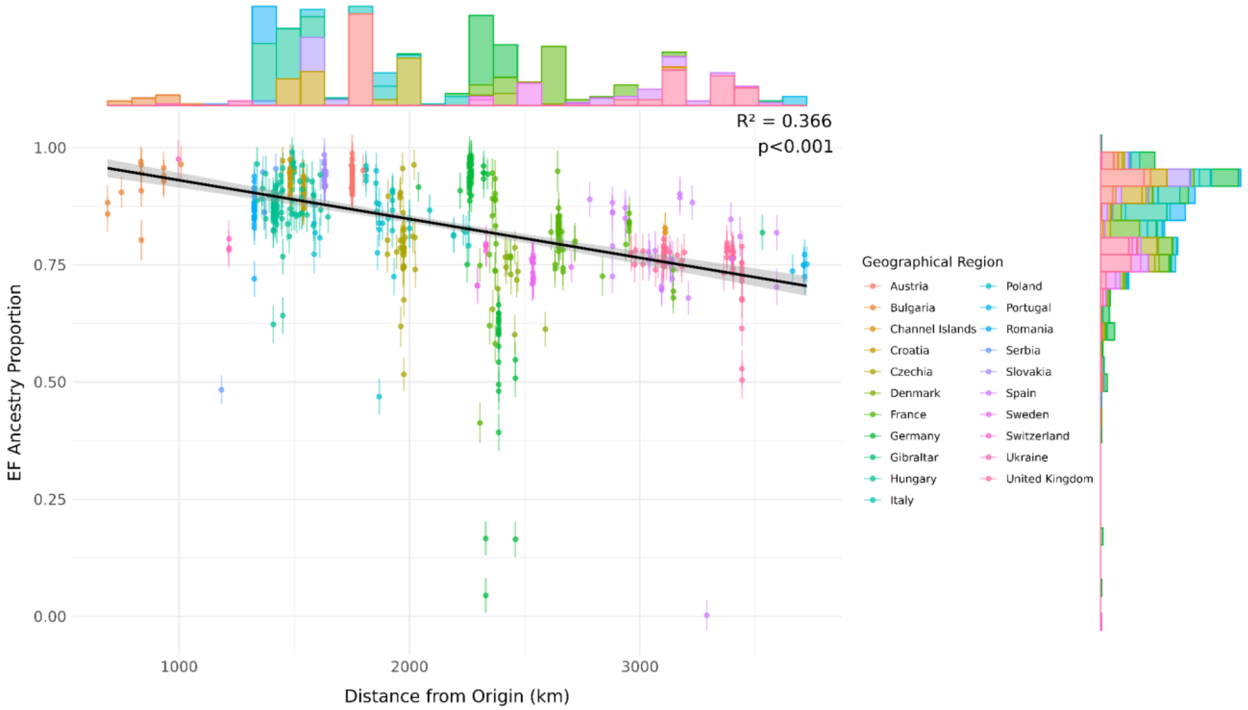

Supplementary Figure 3: EF ancestry proportion estimations from ancient individuals over distance from farming origin and their geographic region

Linear model performed using the 'lmrob' function from the R package 'robustbase'[17] of individual distance from farming origin (x-axis) and their Early Farmer (EF) ancestry estimation (y-axis) (slope = -7.555e-05, R^2 = 0.366, p-value < 0.001, grey shading represents the standard error). EF estimation is shown for individuals with a plausible qpAdm[5] model (p-value ≥ 0.01, admixture weights [0, 1], admixture weight standard error < 0.022, and Steppe ancestry ≤ 0.05). Each point represents the qpAdm[5] EF ancestry estimation for each individual with error bars representing the 95% CI. Points are colored by geographical region/country. Histograms along the sample distance and EF ancestry proportion are plotted on the top and right, respectively. These show the observed counts of points colored by geographical region.

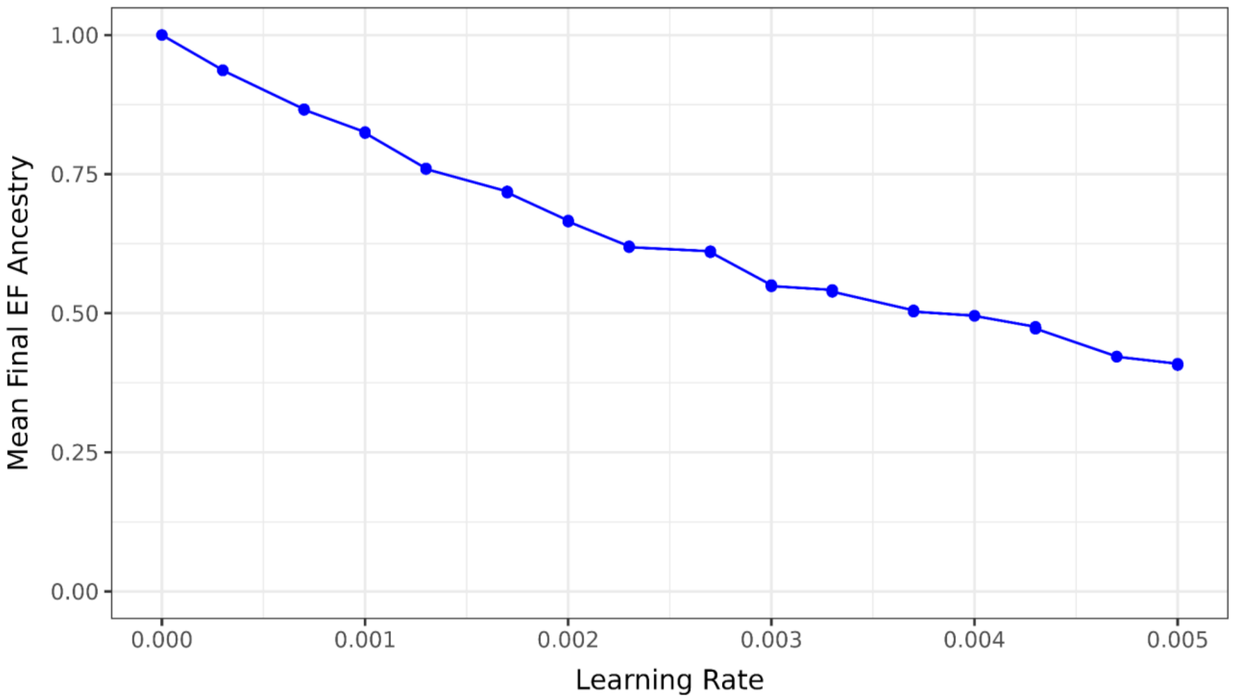

Supplementary Figure 4: Effect of learning rates on remaining EF ancestry
16 simulated learning rates used for fitting empirical data and their effect on the proportion of EF ancestry in the population following the expansion, when farming has become ubiquitous. Figure shows the mean proportion of EF ancestry in the population across the entire landscape. All 16 runs assume fully within-group mating. All runs used the simple square landscape.

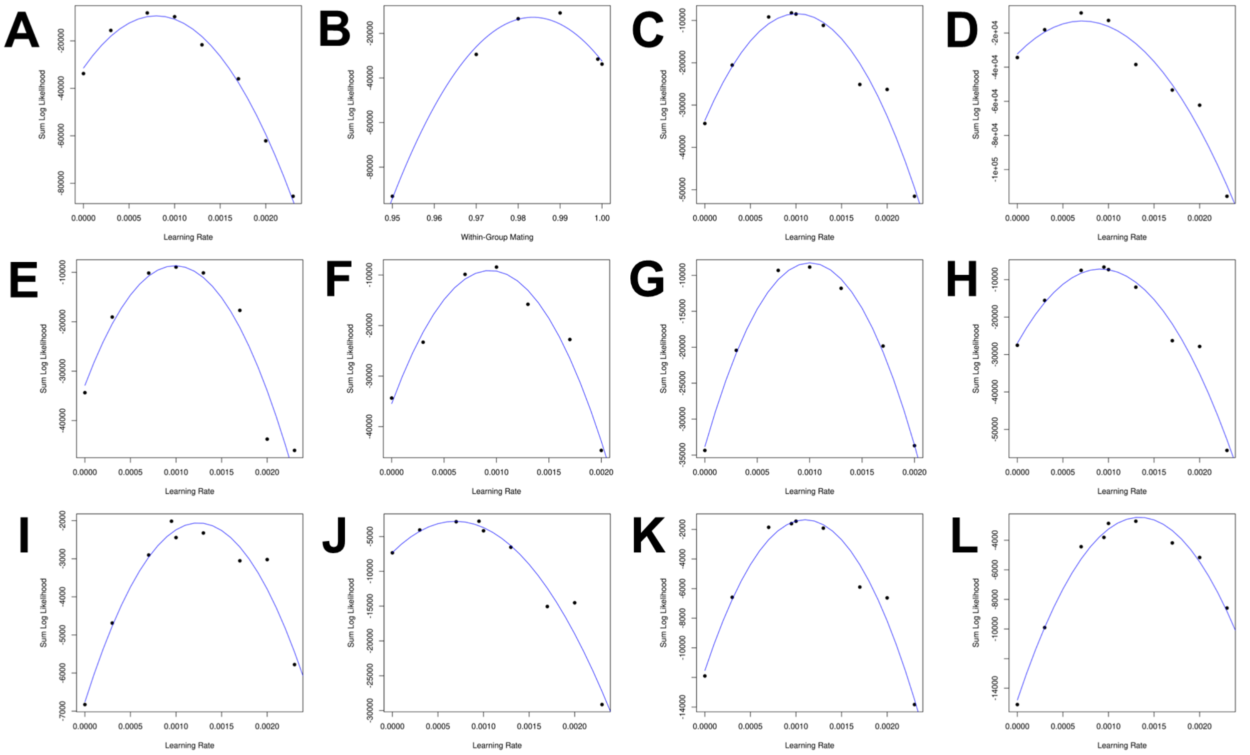

Supplementary Figure 5: Quadratic polynomial fit to the log-likelihood values to determine the maximum likelihood estimates

(A) Learning rate estimates for simple landscape map. (B) Within-group mating estimates for simple landscape map. (C) Learning rate estimates for complex landscape map. (D) Learning rate estimates for complex landscape map with alternative mortality curve. (E) Learning rate estimates for complex landscape map with farmer dispersal biased in the East-West Direction. (F) Learning rate estimates for complex landscape map with topography and small Mediterranean dispersal increase. (G) Learning rate estimates for complex landscape map with topography, Danube/Rhine dispersal increase and larger Mediterranean dispersal increase. (H) Learning rate estimates for complex landscape map fitting Continental Route sim individuals (latitude > 45 degrees) to Continental Route ancient individuals. (I) Learning rate estimates for complex landscape map fitting Mediterranean Route sim individuals (latitude <= 45 degrees) to Mediterranean Route ancient individuals. (J) Learning rate estimates for complex landscape map fitting to Early Neolithic ancient individuals (sample age > 6500 ybp, n=288 individuals). (K) Learning rate estimates for complex landscape map fitting to Middle Neolithic ancient individuals (sample age >= 5500 ybp and Sample Age <= 6500 ybp, n=206 individuals). (L) Learning rate estimates for complex landscape map fitting to Late Neolithic ancient individuals (sample age < 5500 ybp, n=288 individuals). In all cases, we selected 6-9 log-likelihood points close to the optimum to enable a more accurate local fit of the quadratic approximation. All aDNA datasets had a sample size of n=618 individuals unless otherwise specified.

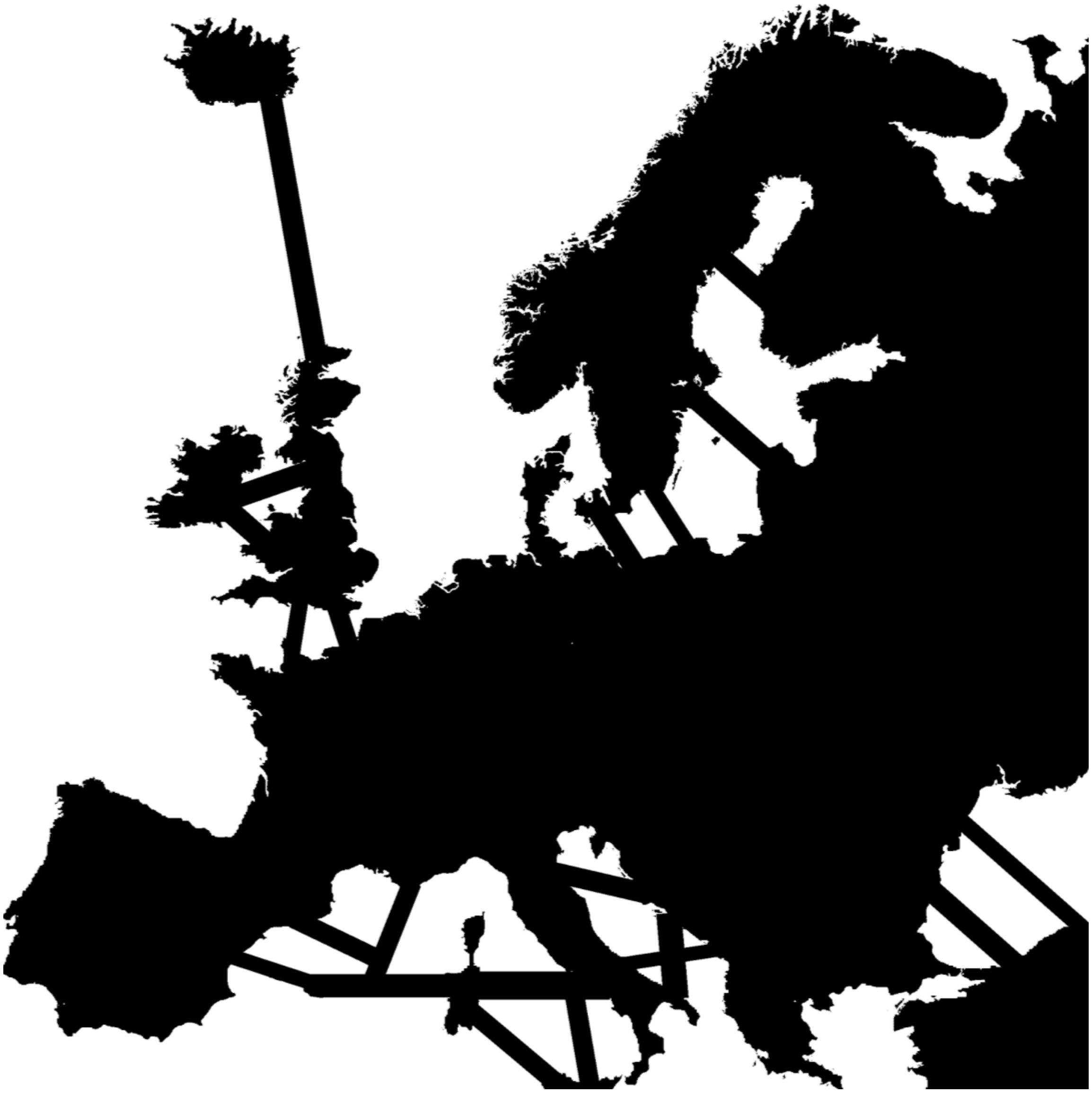

Supplementary Figure 6: 2D landscape map for complex geography simulations
Complex map runs were conducted on this map image which was used to project the boundaries of the simulation landscape on a 2-dimensional X, Y coordinate plane where each unit is 1km. Individuals were only able to exist on black pixels (i.e., individuals were prohibited from living in the ocean). The visible "land bridges" seen in the Mediterranean (and elsewhere) facilitated water crossings that would have taken place via boat, but in the simulation were larger than the possible yearly step size for individuals. Map file adapted from the European Environmental Agency's (EEA) Elevation map of Europe[18], available at the following URL:
(https://www.eea.europa.eu/ds_resolveuid/558D91E1-3DB0-4639-9F70-2012CC4453A5).

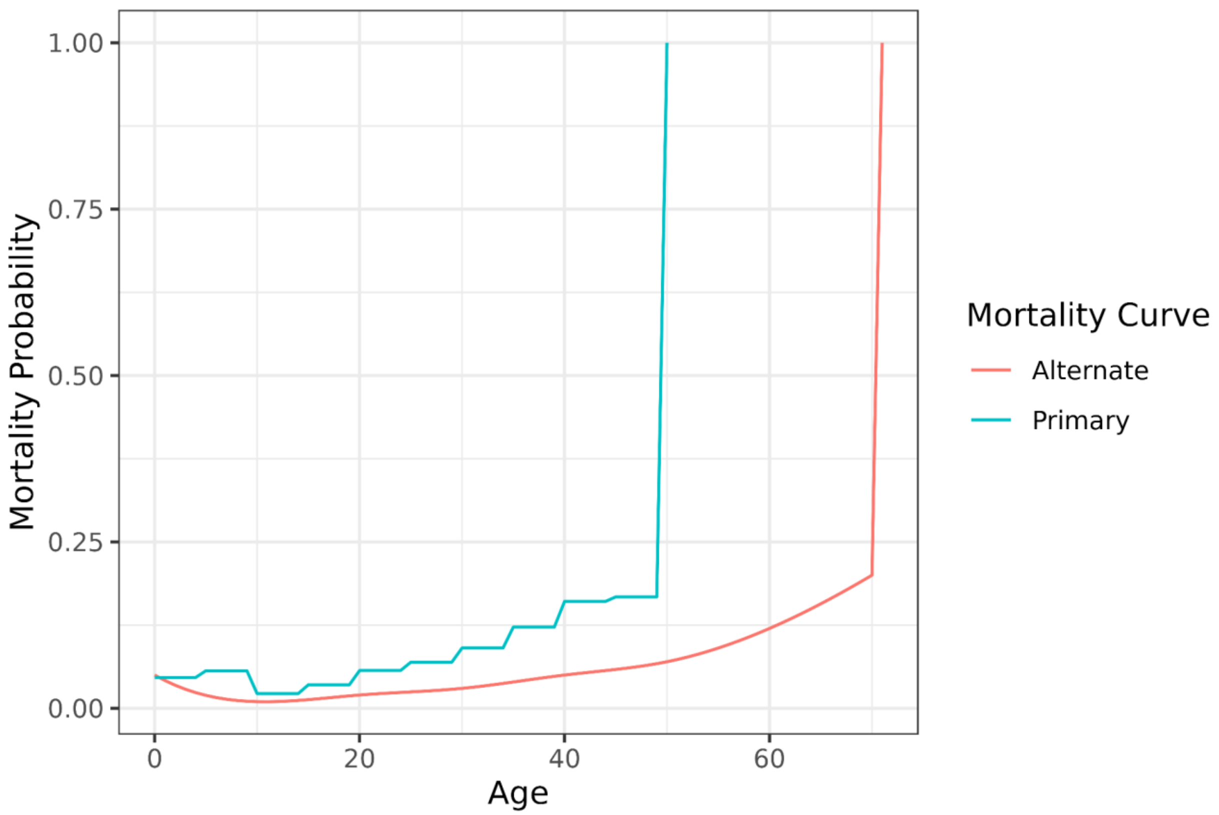

Supplementary Figure 7: Two tested mortality curves
The probability of mortality under two distinct mortality curves (see Supplementary Data File 3; Supplementary Data File 4) based on osteological age at death data[19,20]. The primary mortality curve used in our model is shown in blue and the alternative mortality curve is in red. The primary curve is based on data from Papathanasiou (2005)[19] and the alternative curve is based on data from Eshed et al. (2004)[20].

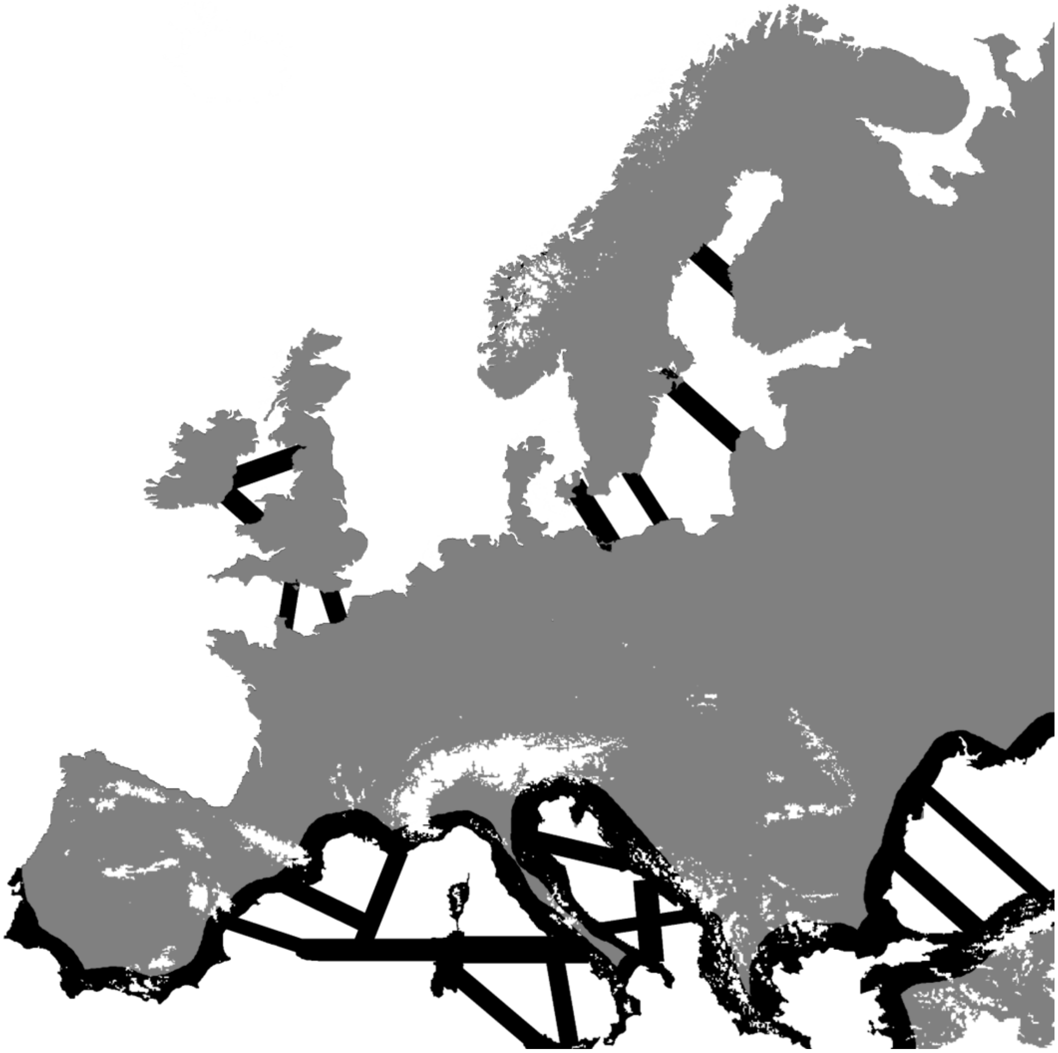

Supplementary Figure 8: 2D Complex landscape map with topography and Mediterranean dispersal increase

Complex map runs with distinct expansion routes featuring complex landscape map with topography and small Mediterranean dispersal increase were conducted on this map image. This is a representation of the two overlaid maps used to project the boundaries of the simulation landscape on a 2-dimensional X, Y coordinate plane where each unit is 1km. Topological features now appear on the map and act as a possible barrier to dispersal. Waterways and coastal routes (back) were overlaid on the topological map. This overlay allowed landscape heterogeneity within the expansion because unlike the simpler simulation models we tested, in this model, individuals' locations on the landscape dictate the possible dispersal distance, for that individual, for that year. Individuals could travel a slightly further distance per year along the coast and waterways and the standard distance per year within the rest of the continent (gray). Map file adapted from the European Environmental Agency's (EEA) Elevation map of Europe[18], available at the following URL:
(https://www.eea.europa.eu/ds_resolveuid/558D91E1-3DB0-4639-9F70-2012CC4453A5).

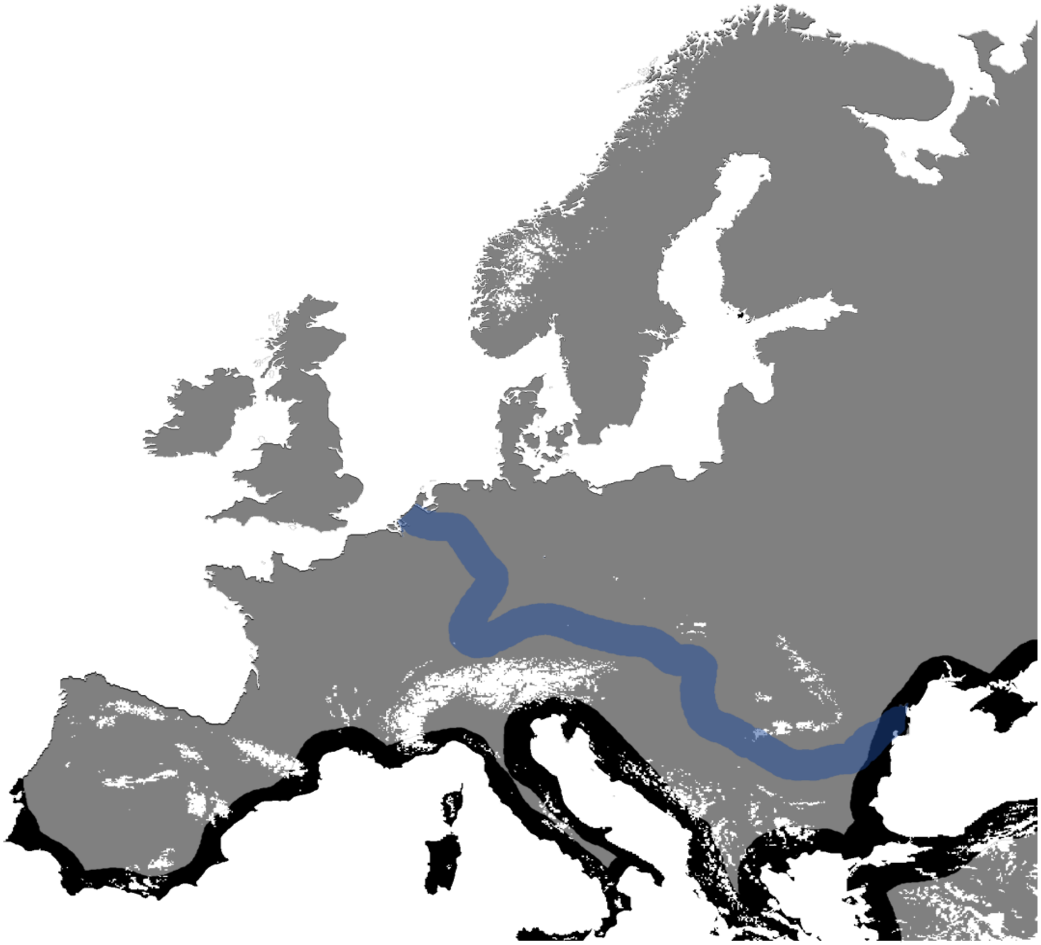

Supplementary Figure 9: 2D Complex landscape map with topography, Danube/Rhine dispersal increase and larger Mediterranean dispersal increase

Complex map runs with distinct expansion routes featuring topography, Danube/Rhine dispersal increase and larger Mediterranean dispersal increase were conducted on this map image. This is a representation of the three overlaid maps used to project the boundaries of the simulation landscape on a 2-dimensional X, Y coordinate plane where each unit is 1km. Topological features now appear on the map and act as a possible barrier to dispersal. Within the simulations, the coastal route (back) and Danube/Rhine route (blue) were overlaid on the topological map. This overlay allowed landscape heterogeneity within the expansion because unlike the simpler simulation models we tested, in this model, individuals' locations on the landscape dictate the possible dispersal distance, for that individual, for that year. Thus, the different routes facilitated different dispersal distances and paths (simulating multiple routes). Individuals could travel the greatest distance per year along the coast, the next largest distance along the Danube/Rhine corridor and then the shortest distance per year within the rest of the continent (gray). The land bridges seen in Supplementary Fig 6 and 8 are no longer necessary in this map, as water travel facilitates longer possible dispersal distance. Map file adapted from the European Environmental Agency's (EEA) Elevation map of Europe[18], available at the following URL:
(https://www.eea.europa.eu/ds_resolveuid/558D91E1-3DB0-4639-9F70-2012CC4453A5).

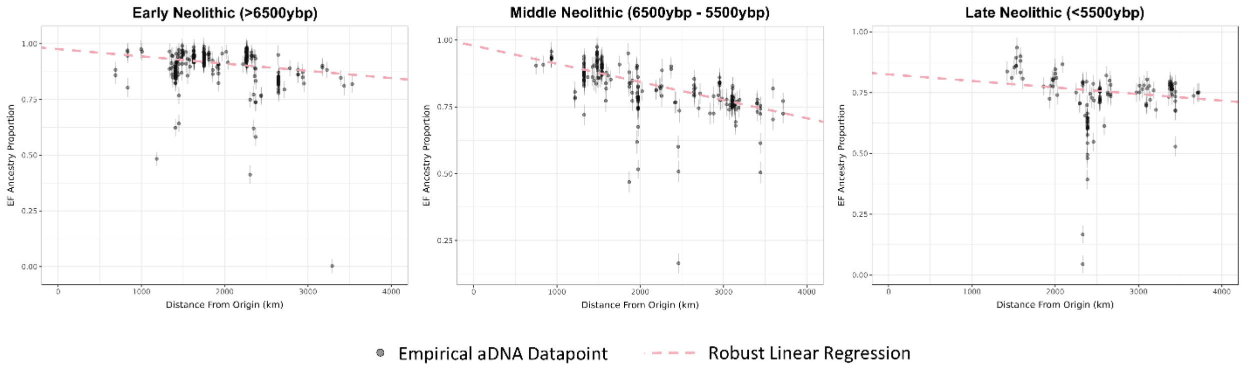

Supplementary Figure 10: EF ancestry estimation in ancient individuals and their distance from origin across time periods

EF ancestry proportion estimations in ancient individuals binned by time period over their distance from farming origin (km). The pink dashed line is a robust linear regression of the aDNA data showing the cline of the EF ancestry with increasing distance from the farming origin (Early Neolithic slope = -3.25032e-05, Middle Neolithic slope = -6.791753e-05, Late Neolithic slope = -2.699051e-05). The regression was performed using the 'lmrob' function from the R package 'robustbase'[17]. We find no significant difference in the slope of the ancestry cline between the Early and Middle Neolithic (p=0.0636) or when comparing Early to Late (p=0.0786).

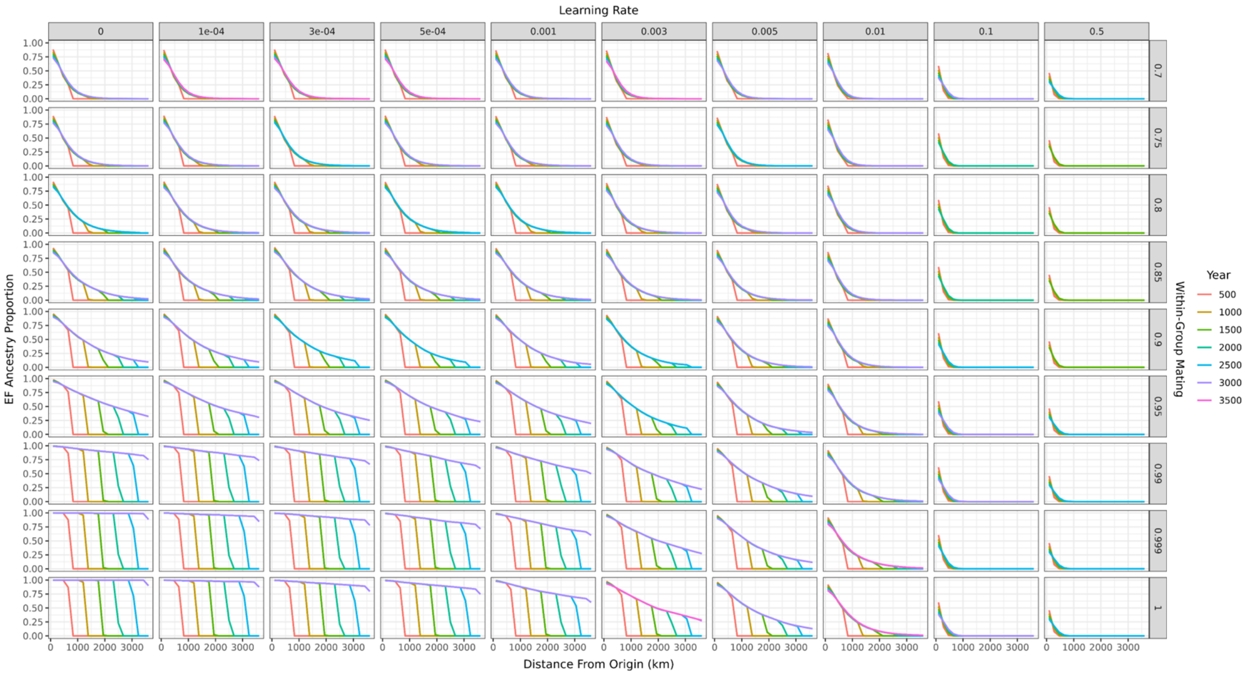

Supplementary Figure 11: Interaction of learning and within-group mating parameters

Y-axes show the EF ancestry proportion in the population through the course of the simulation plotted over distance from origin (km). Lines show the progression of the expansion over time with different colored lines plotted in 500 year increments. Plot faceted on the x-axis by learning rate and on the y-axis by within-group mating parameters tested.

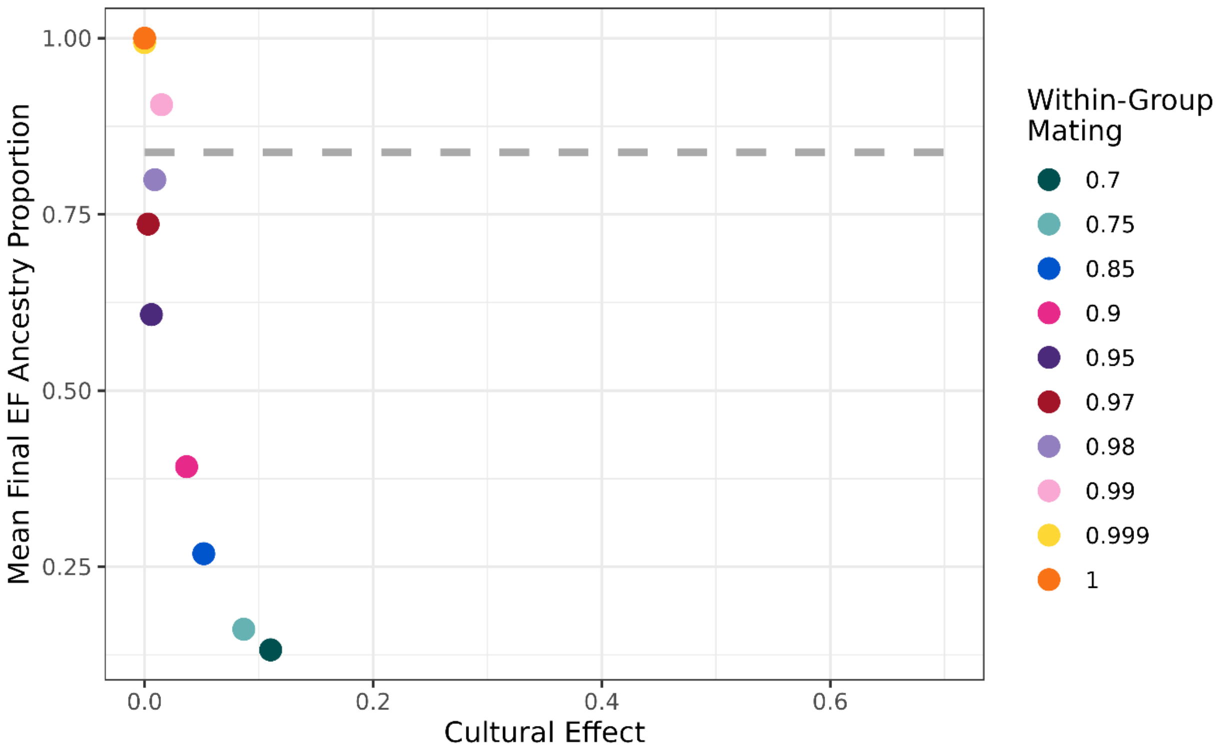

Supplementary Figure 12: Mean final EF ancestry in the population as a function of the cultural effect given various within-group mating rates

Cultural effect describes the percent contribution of cultural transmission to the front speed in addition to a baseline speed of a fully demic model. This figure shows the cultural effect under various within-group mating parameters (as compared to the same plot but for learning rate parameters in main text Fig. 7), and the mean EF ancestry proportion across the simulated population upon the conclusion of the simulation when farming has become ubiquitous (colored points). Simulations were run with no peer-to-peer learning and only parent-to-child vertical cultural transmission via various within-group mating rates (right). For comparison to empirical data, the dashed gray line represents the mean proportion of EF ancestry from our aDNA estimates across the entire landscape.

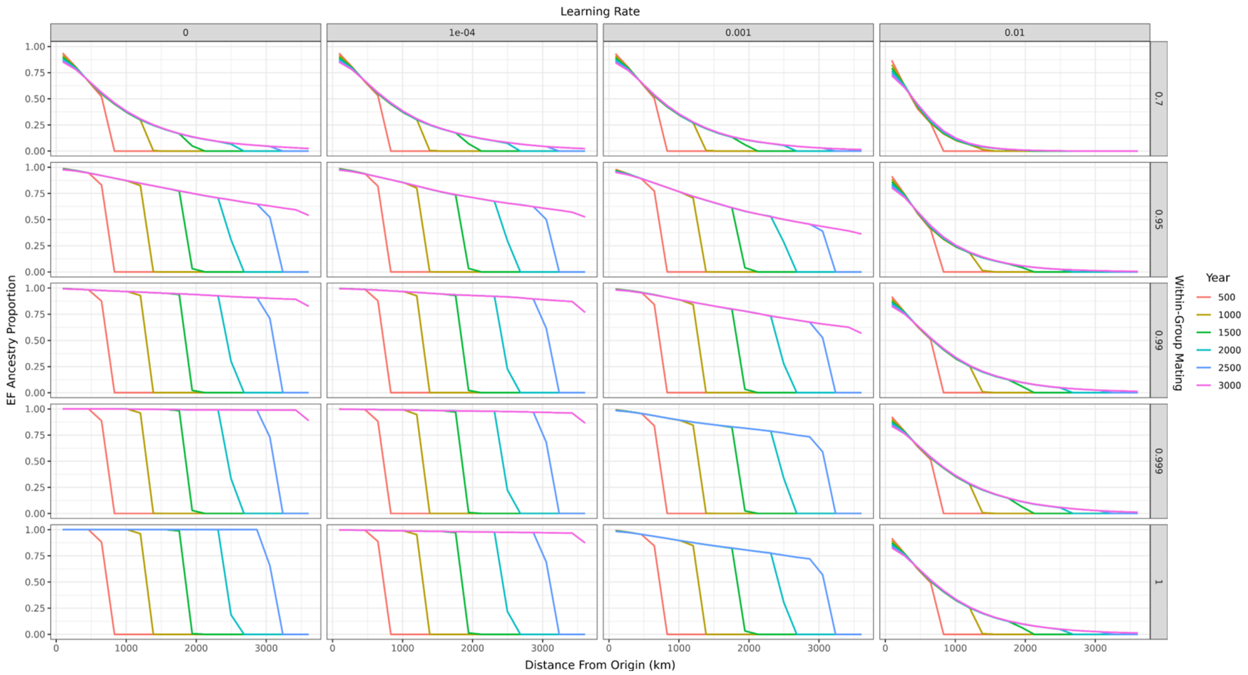

Supplementary Figure 13: Interaction of learning and within-group mating parameters without the assumption that children of farmers only become farmers

These plots are results from simulations where the offspring of a farmer and a HG has a 50/50 chance of becoming either a HG or a farmer. Other simulations in the study assume that any non-within-group matings between HGs and farmers always result in a farming offspring (see Supplementary Figure 11 for comparison). The plots in this present figure do not assume farming offspring. The panels show EF ancestry proportion in the population, over the course of the simulation, plotted over distance from origin (km). Lines show the progression of the expansion over time with different colored lines plotted in 500 year increments. Plot faceted on the x-axis by learning rate and on the y-axis by within-group mating parameters tested.

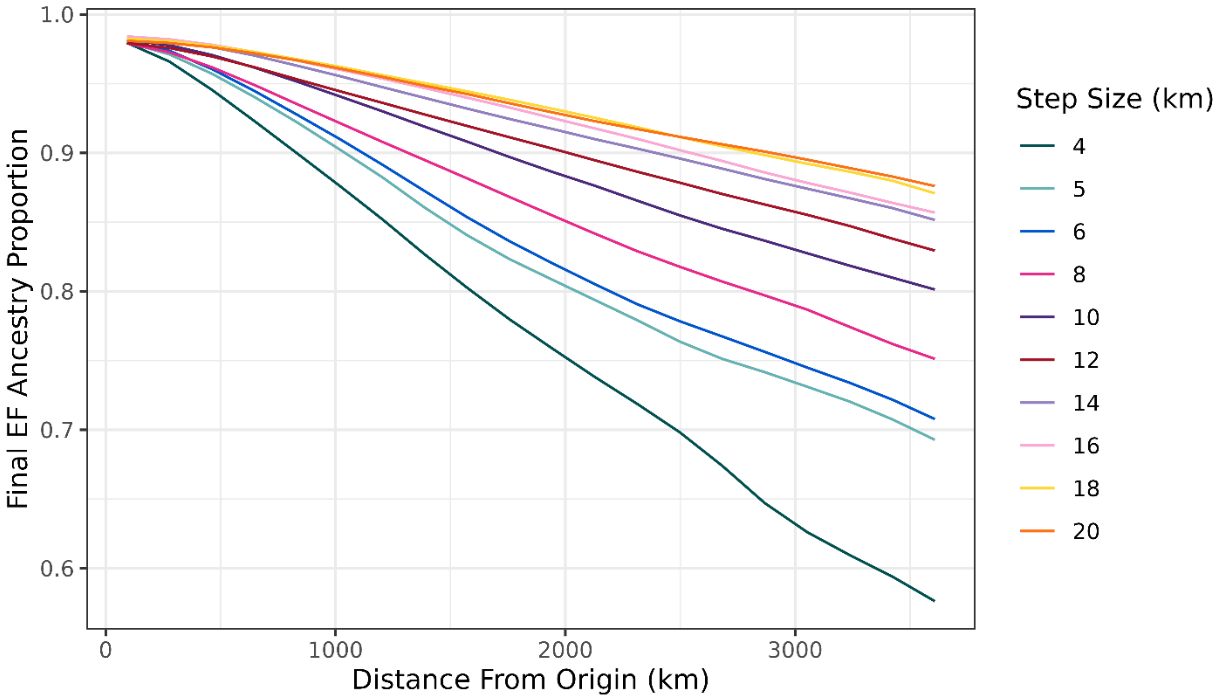

Supplementary Figure 14: Effect of different farmer step sizes on Anatolian farmer ancestry proportion over distance from farming origin

Final EF ancestry proportions plotted over distance from origin (km) upon the conclusion of simulations (when farming is ubiquitous across the landscape). All simulations are run with full within-group mating and a learning rate of 0.001 per year. Each colored line represents a different step size parameter.

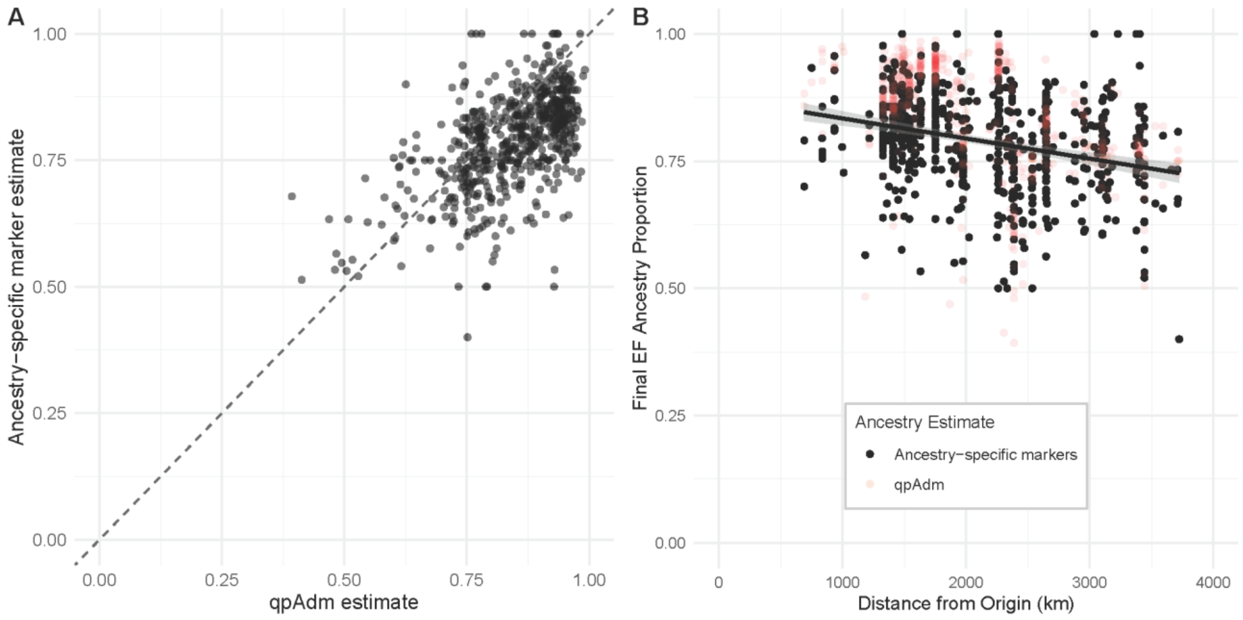

Supplementary Figure 15: Ancestry specific marker estimation comparison with qpAdm[5] ancestry estimation

(A) Correlation between the ancestry estimates from the 53 ancestry-informative markers that are fixed between EF and WHG source individuals and our qpAdm[5]-based ancestry estimates. These marker-based estimates are strongly correlated with qpAdm[5]-based ancestry estimates ($r = 0.56$, $p < 0.001$) (B) The resulting cline of EF ancestry over distance from farming origin yields a nearly identical learning rate estimate (0.00103 per year) to that obtained using qpAdm[5] (0.00798 per year).

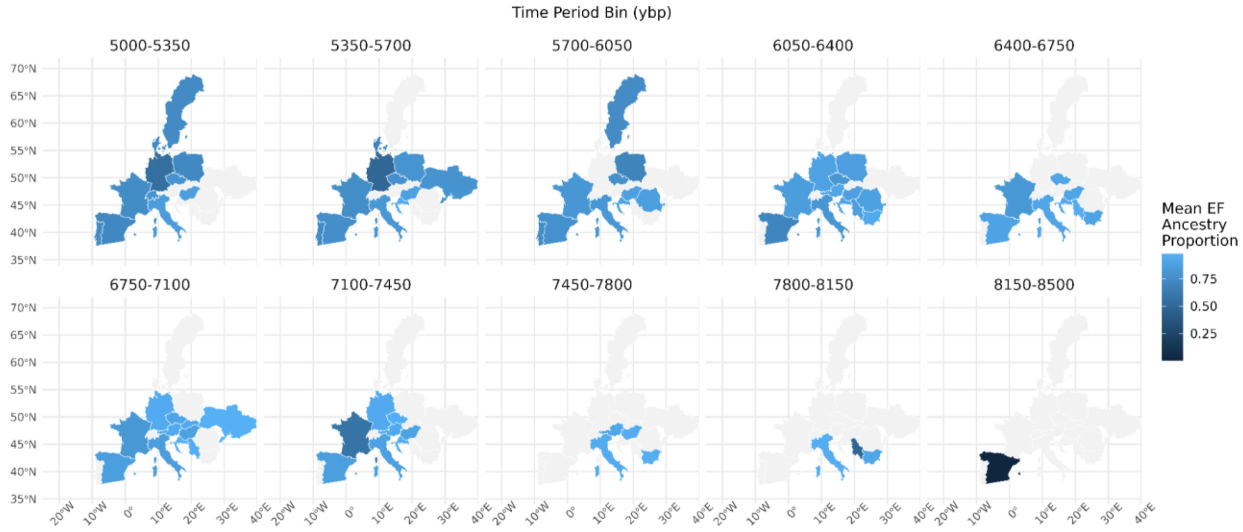

Supplementary Figure 16: Geographical and temporal maps of the average qpAdm[5] EF ancestry estimates calculated for each European country for 10 time bins.

EF ancestry estimates were taken from individuals with a plausible qpAdm[5] model (p-value ≥ 0.01 and admixture weights [0, 1]), admixture weight standard error < 0.022, and Steppe ancestry ≤ 0.05. Grey color indicates no data and blue shade represents the mean EF ancestry proportion with light blue being high and dark blue being low.

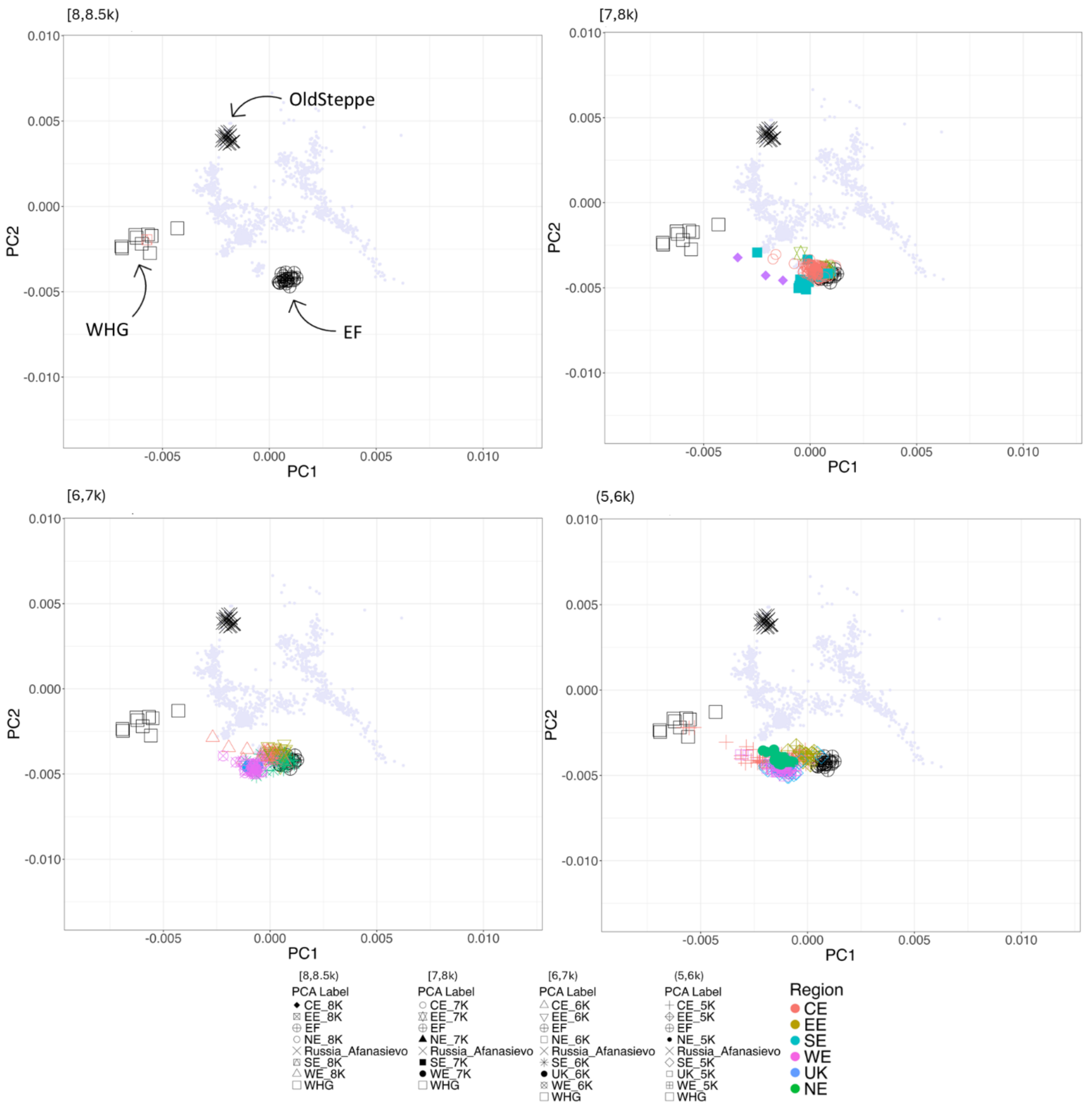

Supplementary Figure 17: Principal component analysis (PCA) of ancestry in ancient individuals used in fitting.

PCA across four temporal bins ([8,8.5k), [7,8k), [6,7k), and (5,6k)) of individuals used in fitting that were filtered for those with a plausible qpAdm[5] model (p-value ≥ 0.01 and admixture weights [0, 1]), admixture weight standard error < 0.022, and Steppe ancestry ≤ 0.05. Individuals used as qpAdm[5] source populations (WHG, OldSteppe, and EF) are included and plotted in black. Analysis individuals are colored by region (CE = central Europe, EE = eastern Europe, SE = southern Europe, WE = western Europe, UK = United Kingdom, and NE = northern Europe). Gray dots are present-day individuals used to form the PCA scaffold, upon which ancient individuals are projected.

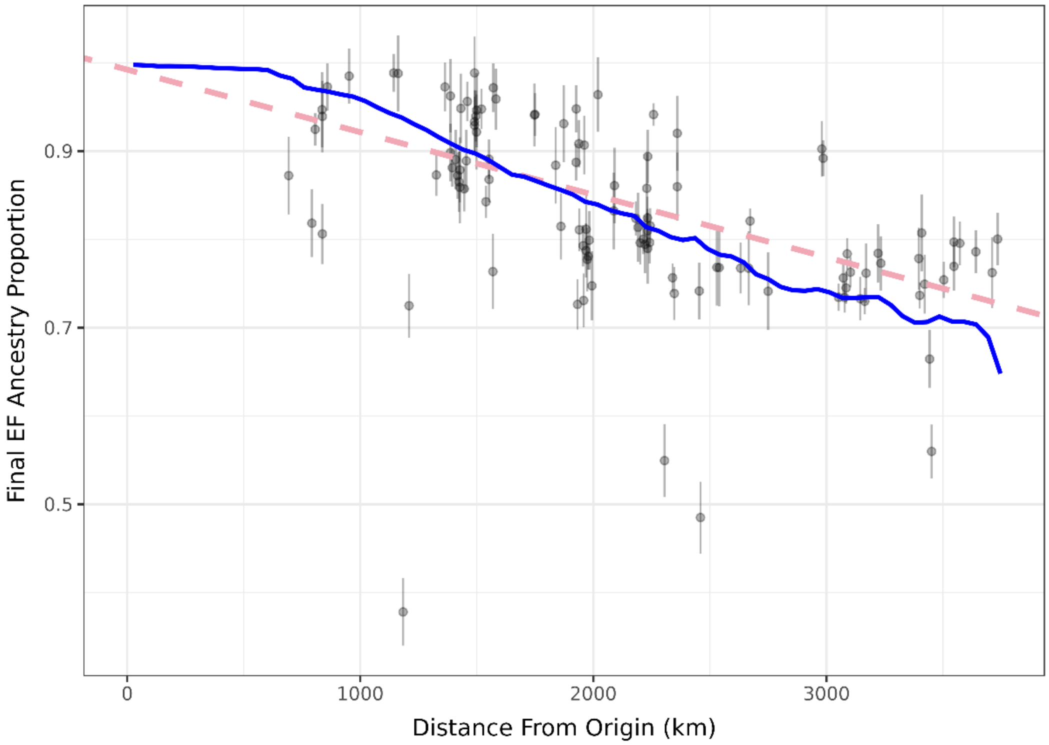

Supplementary Figure 18: Spatial ancestry cline from repeated qpAdm[5] EF ancestry estimation using Anatolian early farmers as a proxy for Neolithic EF ancestry.

Assessment of the robustness of our ancestry estimates to the choice of source population from repeating the qpAdm[5] analysis using Anatolian early farmers (n = 26) instead of Balkan early farmers (n = 21) as the proxy for Neolithic ancestry (as compared to main text Fig. 5b). For this analysis we combined samples from the same site and time into a single estimate of ancestry proportion instead of estimating individual ancestry proportions as we did in main text Fig. 5b. The plot shows empirical aDNA ancestry estimates and our best fitting simulation run from the standard complex landscape (Supplementary Fig. 6) simulation model. The blue line shows the simulated ancestry results for the best-fitting simulation run (learning rate 0.001 per year). Black points show empirical aDNA data points and their qpAdm[5] estimated ancestry proportions, including the 95% CI. The pink dashed line is a robust linear regression of the empirical aDNA data showing the significant negative cline of the EF ancestry with increasing distance from the farming origin (slope = -7.555e-05, R^2 = 0.366, p-value < 0.001). The regression was performed using the 'lmrob' function from the R package 'robustbase'[17].

References

1. Aoki, K., Shida, M. & Shigesada, N. Travelling Wave Solutions for the Spread of Farmers into a Region Occupied by Hunter–Gatherers. *Theor. Popul. Biol* **50**, 1–17 (1996).
2. Aoki, K. Interpreting the demic diffusion of early farming in Europe with a three-population model. *Human Population Genetics and Genomics* **4**, 0010 (2024).
3. Isern, N. & Fort, J. Anisotropic dispersion, space competition and the slowdown of the Neolithic transition. *New J. Phys.* **12**, 123002 (2010).
4. Cortell-Nicolau, A. *et al.* Demographic interactions between the last hunter-gatherers and the first farmers. *Proc. Natl. Acad. Sci.* **122**, e2416221122 (2025).
5. Haak, W. *et al.* Massive migration from the steppe was a source for Indo-European languages in Europe. *Nature* **522**, 207–211 (2015).
6. Villalba-Mouco, V. *et al.* Survival of Late Pleistocene Hunter-Gatherer Ancestry in the Iberian Peninsula. *Curr. Biol.* **29**, 1169-1177.e7 (2019).
7. Olalde, I. *et al.* The genomic history of the Iberian Peninsula over the past 8000 years. *Science* **363**, 1230–1234 (2019).
8. Lipson, M. *et al.* Parallel palaeogenomic transects reveal complex genetic history of early European farmers. *Nature* **551**, 368–372 (2017).
9. Posth, C. *et al.* Palaeogenomics of Upper Palaeolithic to Neolithic European hunter-gatherers. *Nature* **615**, 117–126 (2023).
10. Fernandes, R., Grootes, P., Nadeau, M.-J. & Nehlich, O. Quantitative diet reconstruction of a Neolithic population using a Bayesian mixing model (FRUITS): The case study of Ostorf (Germany). *Am. J. Phys. Anthropol.* **158**, 325–340 (2015).
11. Pawitan, Y. *In All Likelihood: Statistical Modelling and Inference Using Likelihood*.

(Oxford University Press, 2013).

12. Haller, B. C. & Messer, P. W. SLiM 4: Multispecies Eco-Evolutionary Modeling. *Am. Nat.* **201**, E127–E139 (2023).

13. Haller, B. C. & Messer, P. W. SLiM 4.0.1 Manual: An Evolutionary Simulation Framework. (2022).

14. Li, W. & Freudenberg, J. Two-parameter characterization of chromosome-scale recombination rate. *Genome Res.* **19**, 2300–2307 (2009).

15. Pinhasi, R., Fort, J. & Ammerman, A. J. Tracing the Origin and Spread of Agriculture in Europe. *PLOS Biol.* **3**, e410- (2005).

16. Ammerman, A. J. & Cavalli-Sforza, L. L. Measuring the Rate of Spread of Early Farming in Europe. *Man* **6**, 674–688 (1971).

17. Maechler, M. *et al.* robustbase: Basic Robust Statistics. (2024).

18. European Environment Agency. Elevation map of Europe. *eea.europa.eu* https://www.eea.europa.eu/data-and-maps/figures/elevation-map-of-europe.

19. Papathanasiou, A. Health status of the Neolithic population of Alepotrypa Cave, Greece. *Am. J. Phys. Anthropol.* **126**, 377–390 (2005).

20. Eshed, V., Gopher, A., Gage, T. B. & Hershkovitz, I. Has the transition to agriculture reshaped the demographic structure of prehistoric populations? New evidence from the Levant. *Am. J. Phys. Anthropol.* **124**, 315–329 (2004).
