# Supplementary material for: Modeling the European Neolithic expansion suggests predominant within-group mating and limited cultural transmission
Source: Nat Commun. 2025 Aug 25;16:7905. doi: 10.1038/s41467-025-63172-0 (PMC12379212; doi:10.1038/s41467-025-63172-0)
Supplement: Supplementary file 2 — Description of Additional Supplementary Files [file 41467_2025_63172_MOESM2_ESM.pdf]

Description of Additional Supplementary Files

File Name: Supplementary Data File 1

Description: Metadata file for all qpAdm analysis individuals. Provides detailed information for all qpAdm target individuals tested (pre-filtering), the source individuals (EF, WHG, and OldSteppe), and fixed right-groups (OldAfrica, Turkey_N, Russia_Afanasievo, WHGB), including the genetic ID, sample ID, qpAdm analysis ID, publication source, 1240k SNP coverage, data type (capture or shotgun), mean BP date, molecular sex, and site latitude and longitude (total 1675 inds).

File Name: Supplementary Data File 2

Description: qpAdm ancestry estimates from ancient individuals results for all 1531 analyzed individuals pre-filtering. Provides qpAdm results for all target individuals, which were filtered down to 618 European Neolithic individuals that were plausibly modeled as mixtures of WHG and EF ancestry, following the approach outlined by Patterson et al., *Nature*, (2022).

File Name: Supplementary Data File 3

Description: Age-based mortality probability values based on Neolithic osteological age at death data (Papathanasiou, *Am. J. Phys. Anthropol.*, 2005).

File Name: Supplementary Data File 4

Description: Alternative age-based mortality probability values based on Neolithic osteological age at death data (Eshed et al., *Am. J. Phys. Anthropol.*, 2004).

File Name: Supplementary Data File 5

Description: Marker based ancestry estimates for all 618 analyzed Neolithic individuals and 58 source (WHG, EF, OldSteppe) individuals. Provides allelic state of each marker locus and the derived ancestry values for all individuals post-filtering.
