# Supplementary material for: Modeling the European Neolithic expansion suggests predominant within-group mating and limited cultural transmission
Source: Nat Commun. 2025 Aug 25;16:7905. doi: 10.1038/s41467-025-63172-0 (PMC12379212; doi:10.1038/s41467-025-63172-0)
Supplement: Supplementary file 8 — Reporting Summary [file 41467_2025_63172_MOESM8_ESM.pdf]

nature portfolio

Corresponding author(s): Christian D Huber

Last updated by author(s): Jul 15, 2025

Reporting Summary

Nature Portfolio wishes to improve the reproducibility of the work that we publish. This form provides structure for consistency and transparency in reporting. For further information on Nature Portfolio policies, see our Editorial Policies and the Editorial Policy Checklist.

Statistics

For all statistical analyses, confirm that the following items are present in the figure legend, table legend, main text, or Methods section.

n/a	Confirmed	
☐	☒	The exact sample size (*n*) for each experimental group/condition, given as a discrete number and unit of measurement
☐	☒	A statement on whether measurements were taken from distinct samples or whether the same sample was measured repeatedly
☐	☒	The statistical test(s) used AND whether they are one- or two-sided<br>*Only common tests should be described solely by name; describe more complex techniques in the Methods section.*
☐	☒	A description of all covariates tested
☐	☒	A description of any assumptions or corrections, such as tests of normality and adjustment for multiple comparisons
☐	☒	A full description of the statistical parameters including central tendency (e.g. means) or other basic estimates (e.g. regression coefficient) AND variation (e.g. standard deviation) or associated estimates of uncertainty (e.g. confidence intervals)
☐	☒	For null hypothesis testing, the test statistic (e.g. *F*, *t*, *r*) with confidence intervals, effect sizes, degrees of freedom and *P* value noted<br>*Give P values as exact values whenever suitable.*
☒	☐	For Bayesian analysis, information on the choice of priors and Markov chain Monte Carlo settings
☒	☐	For hierarchical and complex designs, identification of the appropriate level for tests and full reporting of outcomes
☐	☒	Estimates of effect sizes (e.g. Cohen's *d*, Pearson's *r*), indicating how they were calculated

Our web collection on statistics for biologists contains articles on many of the points above.

Software and code

Policy information about availability of computer code

Data collection	All scripts used to set up and run the simulations can be found here: https://github.com/TroyLaPolice/European-Neolithic-Expansion<br>The aDNA data were manually curated from publicly available datasets.
Data analysis	All code used to analyze the simulations can be found here: https://github.com/TroyLaPolice/European-Neolithic-Expansion

For manuscripts utilizing custom algorithms or software that are central to the research but not yet described in published literature, software must be made available to editors and reviewers. We strongly encourage code deposition in a community repository (e.g. GitHub). See the Nature Portfolio guidelines for submitting code & software for further information.

Data

Policy information about availability of data

All manuscripts must include a data availability statement. This statement should provide the following information, where applicable:

- Accession codes, unique identifiers, or web links for publicly available datasets
- A description of any restrictions on data availability
- For clinical datasets or third party data, please ensure that the statement adheres to our policy

The Edios code for the SLiM agent-based model used in this study, a file explaining the code for the SLiM agent-based model— as well as landscape files for the model, the R data analysis scripts, and the Mathematica code for the 1D model, can all be found in the following repository: https://github.com/TroyLaPolice/European-Neolithic-Expansion

April 2023

Research involving human participants, their data, or biological material

Policy information about studies with human participants or human data. See also policy information about sex, gender (identity/presentation), and sexual orientation and race, ethnicity and racism.

Reporting on sex and gender	In this research project, we are analyzing ancient DNA data obtained from a publicly available database. Since the samples are derived from individuals who lived millennia ago, they do not involve living human participants.
Reporting on race, ethnicity, or other socially relevant groupings	No living human participant data was used in this study.
Population characteristics	No living human participant data was used in this study.
Recruitment	No living human participant data was used in this study.
Ethics oversight	No living human participant data was used in this study.

Note that full information on the approval of the study protocol must also be provided in the manuscript.

Field-specific reporting

Please select the one below that is the best fit for your research. If you are not sure, read the appropriate sections before making your selection.

☐ Life sciences ☐ Behavioural & social sciences ☒ Ecological, evolutionary & environmental sciences

For a reference copy of the document with all sections, see nature.com/documents/nr-reporting-summary-flat.pdf

Ecological, evolutionary & environmental sciences study design

All studies must disclose on these points even when the disclosure is negative.

Study description	This study explores the dynamics of the Neolithic revolution, a critical period when human societies transitioned from foraging to farming. The central debate in this area involves understanding whether this shift was driven primarily by cultural diffusion (the spread of farming knowledge and practices) or by demic diffusion (the migration and replacement of populations). The study utilizes mathematical models, agent-based simulations, and ancient DNA analysis to dissect the contributions of cultural diffusion and between-group mating to the expansion of farming.
Research sample	We estimated Anatolian ancestry from a dataset of 1531 aDNA samples from the Allen Ancient DNA Resource (AADR). We selected ancient samples that represent populations concurrent with and post the farming expansion but before the subsequent Steppe expansion.
Sampling strategy	From published literature, it is known that there is a cline in Anatolian ancestry across Europe during the early Neolithic. We replicate and estimate the slope of this cline with a sample size larger than available in those previous studies.
Data collection	This study did not collect any data but used a publicly available resource.
Timing and spatial scale	This study did not collect any data but used a publicly available resource.
Data exclusions	We selected ancient samples that represent populations concurrent with and post the farming expansion but before the subsequent Steppe expansion in Neolithic Europe. For ancestry estimation, we only selected samples that allowed accurate ancestry estimation with qpAdm.
Reproducibility	All analyses of this study are fully reproducible with the code at this repository: https://github.com/TroyLaPolice/European-Neolithic-Expansion
Randomization	This is an observational study and thus didn't involve randomization.
Blinding	Given the nature of the study design, blinding during data collection was not applicable. However, data analysis was conducted without prior preference of any specific outcomes associated with the data sets.

Did the study involve field work? ☐ Yes ☒ No

Reporting for specific materials, systems and methods

We require information from authors about some types of materials, experimental systems and methods used in many studies. Here, indicate whether each material, system or method listed is relevant to your study. If you are not sure if a list item applies to your research, read the appropriate section before selecting a response.

Materials & experimental systems

n/a	Involved in the study
☒	☐ Antibodies
☒	☐ Eukaryotic cell lines
☐	☒ Palaeontology and archaeology
☒	☐ Animals and other organisms
☒	☐ Clinical data
☒	☐ Dual use research of concern
☒	☐ Plants

Methods

n/a	Involved in the study
☒	☐ ChIP-seq
☒	☐ Flow cytometry
☒	☐ MRI-based neuroimaging

Palaeontology and Archaeology

Specimen provenance	All ancient DNA data was acquired from the publicly available database: Allen Ancient DNA Resource (AADR) v62.0
Specimen deposition	N/A
Dating methods	Sample dates were obtained from published materials.

☐ Tick this box to confirm that the raw and calibrated dates are available in the paper or in Supplementary Information.

Ethics oversight	No ethical approval was required. All ancestry results were either simulated or reanalyzed from the publicly available database: Allen Ancient DNA Resource (AADR) v62.0

Note that full information on the approval of the study protocol must also be provided in the manuscript.

Plants

Seed stocks	N/A
Novel plant genotypes	N/A
Authentication	N/A
